# Impact of social ties on self reported health in France: Is everyone affected equally?

**DOI:** 10.1186/1471-2458-8-243

**Published:** 2008-07-18

**Authors:** Zoë Heritage, Richard G Wilkinson, Olivier Grimaud, Kate E Pickett

**Affiliations:** 1 Dept of Epidemiology and Public Health, Nottingham University, UK; 2 French School of Public Health (EHESP), Rennes, France; 3 Dept of Health Sciences, University of York, UK

## Abstract

**Aim:**

To examine the association of social ties and income with self reported health, in order to investigate if social ties have a greater impact on the health of people on low incomes compared to those financially better off.

**Methods:**

A nationally representative cross-sectional study of 5205 French adults using data from questionnaires which asked about health, income and relationships with family and friends etc.

**Results:**

Less than good self-rated health (SRH) is twice as frequently reported by people in the lowest income group than those in the highest income group. People with low incomes are also more likely to have felt alone on the previous day, received no phone call during the last week, have no friends, not be a member of a club, and to live alone. Socially isolated people report lower SRH. Likelihood ratio tests for interaction vs. main effect models were statistically significant for 2 of the measures of social ties, borderline for 2 others and non-significant for one. For 4 of the 5 indicators of social ties, larger odd ratios show that social isolation is more strongly associated with less than good SRH among people on low incomes compared to those with a higher income.

**Conclusion:**

Social isolation is associated with 'less than good' self-rated health. This effect appears to be more important for people on a low income.

## Background

Low socioeconomic status (SES) and less income are associated with poor health in France as in other developed countries [1-3]. There is still a debate as to how much this is due to the direct effects of material circumstances [4] to psychosocial factors [5], or to the psychosocially mediated effects of either. Those favouring psychosocial explanations point to the importance of social relations, sense of control etc as health determinants. They also emphasise that middle income people, not affected by material poverty, suffer from poorer health than the richer groups in a society [6,7]. The same has been found to be true in France [8,9].

In 1979, Berkman and Syme [10] showed that mortality increased with a lack of social relationships. The results were still significant after controlling for social class and behaviours such as smoking. Other studies have also shown that the frequency and quality of social relationships are positively associated with health [11-13]. The French 'Gazel' cohort has indicated the negative influence of poor social integration on male mortality [14]. Melchior et al [15] using data from the same cohort, found that a lack of social support and a dissatisfaction with social relations, rather than the size of social network, decreased health. Antonucci and Fuhrer [16,17] found that French older adults with few social network connections had an increased risk of mortality: the age-adjusted rate ratio was 2.69 for men and 1.56 for women.

The exact mechanism linking friendship and social support to health has yet to be established conclusively. Several pathways through which social affiliations could influence health have been identified. Behavioural pathways include smoking, diet etc. Psychological pathways include the effects of social connectedness on feelings of self-esteem and coping. Physiological pathways are the biological processes through which our bodies are affected by social relationships [18]. Oxytocin was originally known as a neuropeptide key for birthing but it now appears to have an important role in the development of all types of social bonding. Dysfunction in this system may lead to autism [19]. Knox & Uvnas-Moberg [20] outline how social support can influence the existence of cardiovascular disease via neuroendocrine pathways such as sympathetic-adrenomedullary influences on blood pressure mediated by the release of oxytocin.

Although social relationships have been associated with good health, as Stansfeld [21] pointed out, "relatively little work has attempted to relate macro-social variables such as social class to social support" page 162 [21]. In the Whitehall II study, higher grade civil servants were in better health and had more friends and received more emotional support than their lower grade colleagues [22].

There is indisputable evidence of the link between SES and health. Most studies looking at social ties do show a positive association with health, but there are ongoing discussions about the exact mechanisms (structural or functional) [23,24]. However, not all of the social ties studies have controlled for SES. The apparent health effects of friendship and other social ties may have a different effect at low and high SES.

The aim of this study is to examine the association of social ties and self reported health across levels of income in France. Our hypothesis is that the impact of social isolation on health will be greatest among the most economically vulnerable i.e. for those people on low incomes.

## Method

The data used in this study were gathered during May 1997 by the National Institute of Statistics, Paris (INSEE, Institut National de la Statistique et des Etudes Economiques) as part of the Permanent Survey of Household Living Conditions (PSHLC). Ethics committee approval was obtained by INSEE for the original survey. The questionnaires covered many different domains. For this study, we used socio-demographic data, plus information about self-rated health (SRH), household income and social relationships.

The PSHLC randomly selected a total of 8,000 households from mainland France and 5,691 (71%) were interviewed. Up to 3 adults per household were asked to respond to questionnaires. We randomly selected one adult per household. Key data (income or SRH) were missing for 5% of individuals. The sample was compared to the census population collected 2 years later. It was found to be representative of the French population by age, gender and region, except for those less than 20 years old who were under represented. We excluded them, giving an analytic sample of 5, 205 respondents aged 20 years and over.

Individuals were classified according to income. The survey asked for the total net annual income for the household in 13 categories. In France, net household income refers to income after all social security/national insurance charges have been deducted but before income tax and local taxes have been paid. To adjust for household size, we followed the conversion used by the Luxembourg Income Study [25] and calculated an equivalised income for each individual by dividing the mean of each income category by the square root of the number of people in the household. This variable was collapsed into 4 categories with approximately equal numbers of individuals in each.

Five measures of social ties were chosen from the questionnaire. Where necessary, replies were transformed into dichotomous variables. The first variable related to a subjective feeling of loneliness. Respondents were asked "Thinking about yesterday, did you have the feeling of being: alone/supported by others/not one or the other". We coded 'feeling alone yesterday' as 1 and the other replies as 0. The following four variables were related to more structural aspects of a social network. The questions were "During the last 8 days, have you had at least one personal telephone conversation with someone who is not a member of your household? Yes/No"; and "Have you friends, men or women, outside your immediate family? Yes/No". A variable for club membership was created from two questions. The first asked "Are you part of an association or similar structure (sports, cultural, scientific, musical or regional traditions club etc)? and the second referred to membership of a school parents association, humanitarian NGO, religious group, political party or trade union. If the respondent said they participated regularly or irregularly to either question, they were classed as a member of a club. We also recorded whether or not the respondent lived alone.

Self-reported health was measured on a 6-point scale. The replies were dichotomised into very good and good (68% of the sample), and average, fair, poor and very poor (32%) which we classified as 'less than good' health.

Self-rated health and social ties were described by income categories and differences in proportions were tested using Pearson chi-square tests. We also estimated logistic regression models for the risk of 'less than good' SRH by each measure of social ties, adjusting for age and sex. Each one of the 5 measures of social ties was examined separately. The number of records available for the logistic regression varied according to the social tie covariate examined. The exact number of missing records was small and can be found in the results table below. We also looked to see if there was an interaction between age or gender and each social tie.

To investigate whether or not social ties had a different effect on SRH by income category, we compared models containing main effects of social ties and income to interaction models which additionally included interactions between the social tie variable and income, using likelihood ratio tests. This process was repeated for each of the 5 social tie variables. In 2 income strata, logistic regression explored the association between 'less than good' SRH and each social tie by high or low income. For simplification, these models were estimated using a dichotomous income variable, with two approximately equal sized categories. The high and medium-high income categories were combined as 'high' income, and medium-low and low were combined into a 'low' income category. The analyses were adjusted for age and sex, and were conducted using Stata v10.

## Results

Table 1 shows the main characteristics for each of the income groups. The proportion of women is significantly greater in the lower income categories. Average age, however, does not differ by income group. There is a clear income gradient in health with the proportion of people reporting less than good health increasing significantly as income decreases. This gradient is robust to adjustment for age and gender. With the high income category as the reference group, the medium-high category has an odds ratio for less than good health of 1.72 (95% confidence limits 1.42 to 2.09), the medium-low income group of 2.48 (95% CI 2.03 – 3.03), and the low income group of 3.72 (95% CI 3.07 – 4.51).

**Table 1:** Characteristics of individuals in each income category

	Income				
	
	High	Medium-High	Medium-Low	Low	
Number of individuals	1284 (24.7)	1445 (27.8)	1131 (21.7)	1345 (25.8)	
Average age (years)	48.1	47.5	48.6	48.6	ns
Woman (%)	663 (51.6)	758 (52.4)	624 (55.2)	827 (61.4)	p < 0.001
Number (%) reporting less than good health	248 (19.3)	410 (28.7)	410 (28.4)	605 (44.9)	p < 0.001
Number (%) without various social ties:					
Felt alone yesterday	99 (8)	120 (8)	128 (11)	237 (18)	p < 0.001
Did not receive a phone call	88 (7)	152 (11)	155 (14)	268 (20)	p < 0.001
Have no friends	93 (7)	166 (11)	162 (14)	244 (18)	p < 0.001
Not a member of a club	665 (52)	941 (65)	784 (69)	1021 (76)	p < 0.001
Lives alone	281 (22)	358 (25)	282 (25)	556 (41)	p < 0.001

**Figure 1:**
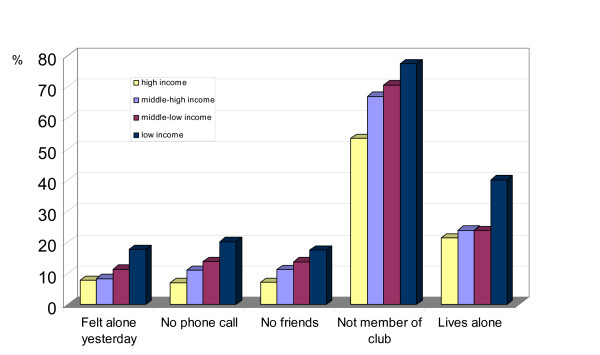
Percentage of people without various social connections by income group.

All 5 measures of social ties are associated with income (see Figure 1 & Table 1). People on lower incomes are more likely to 'feel alone', 'not receive personal telephone calls', 'not have a friend' nor be 'an active member of a club or an association' and 'to live alone'. All these associations are significant (p < 0.001). There is a 2 to 3 fold difference between the highest and lowest income groups for the variables 'felt alone yesterday', 'not receiving a telephone call' and 'not having a friend'.

Table 2 shows that for each of the measures of social ties, those people with strong social ties report better health. In other words, a higher percentage of people who are socially isolated (felt alone, received no telephone call etc) reported 'less than good' health than those with strong social ties. After adjustment for age and gender, odds ratios for less than good health are highly statistically significant (p < 0.001) for 4 of the 5 measures of social ties: felt alone, received no phone call, has no friends and not member of a club.

**Table 2:** Self rated health according to various social ties

	Number (%) reporting very good & good health	Number (%) reporting less than good health	Odds ratio for less than good health [1]	95% conf. limits
**Felt alone yesterday**				
Did not feel alone	3 162 (70.4)	1 331 (29.6)	ref	
Felt alone	272 (46.6)	312 (53.4)	2.42	1.98 – 2.95
missing *123*				

**Phone call**				
Received call(s)	3 090 (69.8)	1 338 (30.2)	ref	
No calls	354 (53.4)	309 (46.6)	1.92	1.60 – 2.32
missing *114*				

**Friends**				
Has friend(s)	3 138 (70.9)	1 285 (29.1)	ref	
No friend	308 (46.5)	355 (53.5)	1.75	1.46 – 2.11
missing *119*				

**Club membership**				
Member of club(s)	1 226 (74.8)	412 (25.2)	ref	
Not member	2 201 (64.5)	1 210 (35.5)	1.72	1.49 – 1.99
missing *156*				

**Other household members**				
Lives with others	2 646 (71.0)	1 082 (29.0)	ref	
Lives alone	877 (59.4)	600 (40.6)	1.09	0.94 – 1.26
missing *0*				

(1) adjusted for age & gender

As the associations between the 5 measures of social ties and SRH were found in a preliminary analysis not to be materially different for men and women (p for interaction from 0.31 to 0.91), all analyses use the combined sample.

The association between 'living alone' and less than good SRH is only seen for the unadjusted analysis (OR 1.67 p < 0.001, 95% confidence intervals 1.47–1.90). Table 2 shows that it is not significant after adjustment for age and sex. People over age 45 were more likely to report poor health if they lived alone (OR = 1.76, CI = 1.50 – 2.07) but the opposite was true for those under 45 years old (OR = 0.82, CI = 0.61 – 1.08). This difference by age between the socially connected and socially isolated who report poor health is not observed among the other 4 measures of social ties.

Table 3 shows the association between social ties and 'less than good' SRH at 2 income levels, high and low. The likelihood ratio test for goodness of fit of the interaction models compared to main effect models, showed that interactions between social ties and income in relation to 'less than good' self reported health were significant for 'no friends' and for 'living alone'; of borderline significance for 'felt alone' and 'no telephone call'; and not significant for 'no club membership'. After adjusting for age and gender, table 3 shows that having a lack of social ties is consistently more strongly associated with poor health at low income than high income. Amongst those with above average income, only the odds ratios of 'less than good' SRH with 2 social tie variables, 'felt alone' and 'not a club member', are significant and then only at the 0.01 level. In the low income stratum, highly significant associations (p < 0.001) are observed between 'less than good' SRH and 4 of the social ties variables: felt alone, received no phone call, has no friends and not a member of a club.

**Table 3:** Association of lack of social ties & less than good self rated health by level of income

	**High income**		**Low income**		**lr test for interaction (1)**
		
	Odds ratio (1)	95% C I	Odds ratio (1)	95% C I	
Felt lonely yesterday	**1.80**	1.30 – 2.49	**2.46**	1.90 – 3.20	**0.06**
Received no phone call	**1.31**	0.95 – 1.89	**1.94**	1.52 – 2.47	**0.07**
Has no friends	**1.17**	0.87 – 1.59	**1.95**	1.52 – 2.49	**0.02**
No club membership	**1.42**	1.16 – 1.74	**1.72**	1.38 – 2.14	**0.80**
Lives alone	**0.84**	0.67 – 1.05	**1.14**	0.94 – 1.39	**0.02**

(1) adjusted for age & gender

## Discussion

Our results confirm that in France, like other developed countries, a health-income gradient exists with poor self reported health being more than twice as frequent among people in the lowest income quartile compared to those in the highest income quartile.

People on a low income are also more likely to have 'felt alone yesterday', 'received no phone call in the last week', 'have no friends', 'not be a member of a club', and to 'live alone'. Febre and Muller [26] found a similar association between income and membership of a club or voluntary organisation in France. They found that only 32% of people in the lowest income quartile were members of a club compared to 57% of those in the highest income quartile.

Our study supports the findings of other authors [10-13,16,17,19,20] that social relationships are closely associated with health. We found that self-reported health is systematically lower among those with lower levels of social contact and integration. The models stratified by income show effects in the direction of our initial hypothesis: the impact of social ties on health is greatest for the most economically vulnerable – i.e. those on a low income. However, the interactions with income are statistically significant only for 2 variables, 'having no friends' and 'living alone' and of boarder-line significance for another 2.

These findings are similar to those of Antonucci et al [27] where vulnerability was measured by low educational attainment. Among people over age 40, they found that less education was generally associated with smaller social networks in Detroit, USA. Further sub-group analyses showed that men with less education, but large social networks and high perceived support, reported health as good as well educated men. This suggests social relationships may protect the health of men with low SES.

Our measure of living alone appears to have a different relationship with health than the other measures of social ties. It is much more affected by an adjustment for age than the other 4 measures of social ties. Further investigation showed that people over age 45 were more likely to report poor health if they lived alone than their younger counterparts. Young people who live alone may do so by choice and therefore would not suffer negative effects on their health.

The psychological measure 'felt alone yesterday' had a stronger association with health (OR = 2.42) than variables measuring the size of social networks (not having friends OR = 1.75 or not being a member of a club OR = 1.72). This confirms other findings that the level of satisfaction with social relationships may be more important for health than indicators relating to the structure of a network [17,18].

Our study indicates that people on low incomes have fewer social ties. Not having much money may prevent people from joining clubs. However a recent study [26] showed the average club/voluntary organisation membership fee in France was only 30€ (£22) per year. Also it seems unlikely that lack of money would prevent people in the middle income groups from having friends or receiving phone calls.

Being on a low income and having few social contacts appears to be particularly associated with poor health. People on low incomes may lack the social confidence and the self-esteem necessary to join clubs, telephone friends etc [6,7]. This population could be biologically stressed due to their low social status, and lack of neuropeptides linked to social bonding, may add to this stress [19,20].

One of the limitations of this study is that although SRH has been shown to be both a robust health indicator and related to mortality [28-30], little is known about how individuals arrive at their SRH replies [31]. Questionnaires about social capital or social ties also have the weakness that someone feeling depressed may rate their number of friends and other social relationships as lower than they really are. This could lead to a reporting bias that only longitudinal studies, with measures of mental health, can address.

Another limitation is the cross-sectional study design, which means that results can only show associations, which may not be causal. Similarly it is not possible to draw conclusions about possible reverse causality, which might occur if people in very poor health became unable to maintain social ties. This seems unlikely, as further analysis of our self reported health variable shows those with 'good' or 'average' health report less social contact than those reporting 'very good' health. Also, cohort studies which by their nature can avoid the problems of reverse causality, have found similar results to ours [10,14,15].

Reverse causality may also occur when sick people lose their job and move down the income scale. In France, however, strong job protection legislation and social insurance helps to prevent ill health reducing income. Education is an important measure of social economic status as, once in employment, education attainment is not affected by poor health. The original questionnaire did contain information about education but the large number of potential reply categories meant it was difficult to interpret the results. Education was also found to be highly related to age with the mean age of primary school/no qualifications group being 62 years compared to 45 years for those with higher secondary or better qualifications. It could be that by using income as a measure of social economic status, we may have primarily looked at the interaction between material deprivation and social ties on health. We have tried to avoid this by using data from the whole sample not just comparing the extremes (middle income people are probably not materially deprived). Also, it should be noted that our findings are very similar to those reported by Antonicci et al [27] who used education attainment as a measure of SES.

It may be that health behaviours are mediators of any relationship between social ties and health, for example, people who are socially isolated may drink or smoke more, and it is possible that these effects are stronger in low income groups. No data were collected in this study on alcohol consumption or diet, but questions were asked about current smoking. Unsurprisingly, smoking prevalence was higher among the low income group. However, when we included smoking in models examining the effect of social ties on SRH, the effects were only slightly attenuated and there were no changes in the statistical significance of the associations.

## Conclusion

This study shows that people on low incomes report less social contact and also poorer health. The effect of lack of social ties on health appears to be of greater magnitude among people on low incomes, compared to those who are better off. If further research confirms this finding, it would reinforce calls to promote public health initiatives that aim to strengthen social ties and social cohesion in economically poor neighbourhoods.

## Consent

No ethical consent was required for this study. The authors only analysed anonymous information.

## Competing interests

The authors declare that they have no competing interests.

## Authors' contributions

ZH & RGW conceived the study. OG & KEP advised on statistical methods. ZH performed the statistical analysis and wrote the first draft. All authors contributed to its revision and approved the final manuscript.

## Pre-publication history

The pre-publication history for this paper can be accessed here:


